# Efficiency of pH-Sensitive Fusogenic Polymer-Modified Liposomes as a Vaccine Carrier

**DOI:** 10.1155/2013/903234

**Published:** 2013-02-04

**Authors:** Shinobu Watarai, Tana Iwase, Tomoko Tajima, Eiji Yuba, Kenji Kono

**Affiliations:** ^1^Division of Veterinary Science, Graduate School of Life and Environmental Sciences, Osaka Prefecture University, Izumisano, Osaka 598-8531, Japan; ^2^Department of Applied Chemistry, Graduate School of Engineering, Osaka Prefecture University, Sakai, Osaka 599-8531, Japan

## Abstract

The usefulness of pH-sensitive fusogenic polymer-(succinylated poly(glycidol)-(SucPG-) modified liposomes as a vaccine carrier in the induction of immune responses was evaluated. Mice were intraperitoneally immunized with ovalbumin- (OVA-) containing SucPG-modified liposomes. After immunization, significant OVA-specific antibodies were detected in the serum. When sera were analyzed for isotype distribution, OVA-specific IgG1 antibody responses were noted in mice immunized with OVA-containing polymer-unmodified liposomes, whereas immunization with OVA-containing SucPG-modified liposomes resulted in the induction of OVA-specific IgG1, IgG2a, and IgG3 Ab responses. In spleen lymphocytes from mice immunized with OVA-containing SucPG-modified liposomes, both IFN-**γ**-(Th1-type-) and IL-4-(Th2 type-) specific mRNA were detected. Moreover, substantial production of IFN-**γ** and IL-4 was demonstrated in spleen cells from OVA-containing SucPG-modified liposomes *in vitro*. These results suggest that the pH-sensitive fusogenic polymer-(SucPG-) modified liposomes would serve effectively as an antigen delivery vehicle for inducing Th1 and Th2 immune responses.

## 1. Introduction

 Immune mechanisms that control diseases include mainly the induction of neutralizing antibodies (humoral immunity) and generation of T cells (cell-mediated immunity), including CD4^+^ helper (Th) and CD8^+^ cytotoxic (cytotoxic T lymphocyte) responses [1, 2]. The success of vaccines depends on two key aspects: identification of specific antigenic targets and the ability to evoke a strong and appropriate immune response. In addition, efficient vaccination strategies have been desired for overcoming new pathogens and for evolution of resistance of microorganisms. Thus, new adjuvants and carriers are essential to this aim, and efficient vaccine delivery systems have been required for the achievement of protective immunity.

 Bilayer vesicles composed of amphiphilic phospholipids (liposomes) have been used as delivery systems for a wide variety of biologically active substance to specific tissues and have also been used as immunological adjuvants to enhance the immune response to several bacterial and viral antigens [3]. In particular, since the liposome-entrapped materials are protected from enzymatic attack until they reach the target sites, the potential usefulness of liposomes as carriers and adjuvants for developing topical and mucosal vaccines has attracted considerable interests during the last few years [4]. Their potential as adjuvants has been demonstrated in several studies, in which the use of liposome-associated antigens resulted in protective immunity [5–14]. From these previous studies, it emerges that the adjuvant effect of the liposomes depends on their physicochemical properties and may be related to prolonged release and protection of encapsulated antigen against the environment and enhanced uptake by the dendritic cells (DCs). It is generally accepted that cationic liposomes are more potent adjuvants compared to anionic and neutral liposomes [14]. Their adjuvant effect has been attributed to several mechanisms, such as nonspecific cell damage (inducing inflammation) at the site of injection, formation of an antigen depot, and improved antigen uptake by DCs through electrostatic interaction between the cationic liposomes and negatively charged groups on the surface of DCs. However, effective delivery of antigenic proteins into cytosol of DCs is important to induce protective immunity. Thus far, numerous attempts have been undertaken to achieve delivery of antigens into the DC's cytosol [15–17]. These nanoparticles might be taken up by DC via endocytosis and enhance the transfer of their encapsulated antigen molecules from endosome and/or lysosome to cytosol by destabilization of the membranes of these acidic compartments through hydrophobic or electrostatic interactions [16, 17]. One of the most effective strategies for efficient introduction of antigenic proteins into cytosol of DC would be to use membrane fusion for liposomes. To date, viral fusion proteins have been used frequently to provide liposomes with fusion ability [18, 19]. Indeed, viral fusion protein-incorporated liposomes have been used to introduce encapsulated antigenic OVA into DC's cytosol and induced efficient cellular immunity [18, 19]. However, viral proteins might provoke unexpected immune responses. Therefore, the use of synthetic carriers might be preferred for the delivery of antigens into DCs.

 To establish effective vaccine delivery system for the induction of protective immunity, we have developed pH-sensitive liposomes, which generate fusion ability under weakly acidic conditions, by surface modification of liposomes with pH-sensitive fusogenic polymer having carboxyl groups, such as succinylated poly(glycidol) (SucPG) [20]. This pH-sensitive fusogenic liposomes encapsulating ovalbumin (OVA) could introduce their contents efficiently into the cytosol of dendritic cells [20]. However, relatively little data on their potential vaccine carrier is inconclusive. 

 To know the usefulness of pH-sensitive fusogenic polymer-modified liposomes as a vaccine carrier, OVA-containing SucPG-modified liposomes were intraperitoneally inoculated to mice, and immune responses were evaluated. We provide here evidence for the induction of strong antigen-specific Th2 (humoral) and Th1 (cell-mediated) immunity. 

## 2. Methods

### 2.1. Materials

 Dipalmitoylphosphatidylcholine (DPPC), dioleoylphosphatidylethanolamine (DOPE), monophosphoryl lipid A (MPL), and ovalbumin (OVA) (SIGMA) were commercial products. Succinylated poly(glycidol) (SucPG) was prepared as previously reported [21, 22]. Molar percentages of glycidol/carboxylated glycidol/n-decylamine-attached units in the resultant SucPG polymer were determined by ^1^H NMR to be 18/74/8 and 9/89/11, respectively [20].

### 2.2. Animals

 Female BALB/c mice (6 weeks old) were purchased from Charles River Japan, Tokyo, Japan. Mice were maintained according to the Standards Relating to the Care and Management of Experimental Animals of Japan. The experiments were carried out in accordance with the guidelines for animal experimentation of Osaka Prefecture University.

### 2.3. Preparation of Liposomes

 Polymer-(SucPG-) modified liposomes that entrap OVA were prepared by the following method. DPPC (4 *μ*mol), DOPE (4 *μ*mol), MPL (16 *μ*g), and SucPG polymer (lipids/polymer = 7/3, w/w), each dissolved in an organic solvent, were mixed in a conical flask. The lipids were dried on a rotary evaporator, and left to stand for 30 min in a high vacuum in a desiccator. After the addition of 1 mL of PBS containing OVA (5 mg/mL) and the incubation at an appropriate temperature for 3 min, the lipid film was dispersed by vigorous vortexing. Any unencapsulated OVA was removed by repeated centrifuging at 14,000 ×g for 20 min at 4°C in PBS, and the resulting liposome suspension was used for immunization.

 Polymer-unmodified liposomes that entrap OVA were prepared from lipid mixture solution containing DPPC (4 *μ*mol), DOPE (4 *μ*mol), and MPL (16 *μ*g) as stated above.

 The amount of OVA entrapped in liposomes was determined by the following method. Ninety *μ*L of isopropyl alcohol was added to a 10 *μ*L suspension of liposome-entrapped OVA (at 3-fold dilution in PBS), followed by vortex mixing. The protein concentration of the resulting solutions was determined using a Bio-Rad protein assay kit (Bio-Rad Laboratories), with bovine plasma gamma globulin used as a standard.

### 2.4. Immunization of Mice

 Mice were divided into 3 groups (5 mice per a group). Each group was intraperitoneally immunized as follows: group I, OVA alone (100 *μ*g protein/100 *μ*L into peritoneal cavity); group II, polymer-unmodified liposomes that entrap OVA (100 *μ*g protein/100 *μ*L into peritoneal cavity); group III, SucPG-modified liposomes that entrap OVA (100 *μ*g protein/100 *μ*L into peritoneal cavity). Two weeks later, mice were boosted with the same immunogen at an equivalent dose. Seven days after secondary immunization, the mice were killed and sera and spleens were harvested. Sera and spleen collected were used for antibody assay and RNA isolation, respectively. Spleen cells were isolated from mice in group III as described previously [23] and used for cytokine measurements.

### 2.5. Antibody Assay

 OVA was diluted with PBS (10 *μ*g protein/mL) and dispensed in 50 *μ*L/well into a 96-well microtiter plate (ASAHI TECHNO GLASS), followed by leaving overnight at 4°C. The plates were washed 5 times with PBS containing 0.1% Tween 20 (washing solution). The wells were treated with 100 *μ*L of PBS containing 1% BSA (solution A), incubated at 37°C for 60 min to block nonspecific binding, and then washed 5 times with the washing solution. After that, 50 *μ*L of sera diluted with solution A were added to each well. The plates were incubated for 60 min at 37°C and washed 5 times with the washing solution, and then 50 *μ*L of horseradish peroxidase-labeled anti-mouse IgA (1 : 2,000 dilution in solution A; American Qualex), IgG (1 : 2,000 dilution in solution A; American Qualex), IgE (1 : 2,000 dilution in solution A; Bethyl Laboratories), IgG1 (at 1 : 1,000 dilution in solution A; Zymed Laboratories), IgG2a (at 1 : 1,000 dilution in solution A; Zymed Laboratories), or IgG3 (at 1 : 1,000 dilution in solution A; Zymed Laboratories) solution was added as the second antibody. Following incubation for 60 min at 37°C, the plates were washed 5 times with the washing solution, and 100 *μ*L of **ο**-phenylenediamine dihydrochloride substrate solution (Sumitomo ELISA Color Reagent Kit; Sumitomo Bakelite) was reacted for 15 min at room temperature. The enzyme reaction was stopped by adding a stopping solution (Sumitomo ELISA Color Reagent Kit), and absorbance at 490 nm was measured with a microplate reader (Model 450, Bio-Rad Laboratories). Antibody titers are represented as the reciprocal of endpoint dilution exhibiting an optical density more than 2.5 times that of the background. 

### 2.6. RNA Isolation from Spleen and Cytokine RT-PCR

 Total RNA was extracted from homogenized spleen tissue using TRIzol reagent (GIBCO-BRL) according to the manufacturer's instruction. The final RNA pellet was resuspended with diethylpyrocarbonate-treated distilled water, and absorbance at 260 nm was measured. Five micrograms of RNA were used for cDNA synthesis using SuperScript II RNase H^−^ reverse transcriptase and oligo-dT_(12–18)_ primer (GIBCO-BRL), according to the manufacturer's instruction. Primers used are shown in Table 1. PCR was performed for each cytokine gene in a 50 *μ*L reaction mixture containing 1 *μ*L of the cDNA, 10 pmol of each primer, 0.2 mM dNTP mixture, 1.5 units of Taq DNA Polymerase (GIBCO-BRL), 1.5 mM MgCl_2_, 20 mM Tris-HCl (pH 8.4), and 50 mM KCl with 5 min denaturation at 94°C followed by 35 cycles consisting of 45 sec denaturation at 94°C, 45 sec annealing at 60°C, and 2 min extension at 72°C. The final extension was 7 min. To rule out contamination of DNA in the RNA preparation, cDNA was prepared by the same procedure without the addition of reverse transcriptase, and PCR was performed. As negative control, water was used as template and as a positive control a cDNA prepared from RNA extracted from PWM-stimulated mouse spleen cells was used. The PCR products were electrophoresed through a 2% agarose gel and stained with ethidium bromide.

### 2.7. Cytokine Measurements

 Spleen cells from nontreated control mice and mice in group III were cultured at a density of 1 × 10^6^ cells/mL with 5 *μ*g/mL of OVA to detect antigen-specific T cell-derived cytokine production. Culture supernatants were collected 5 days after incubation, and the levels of Th1 and Th2 cytokines (IFN-*γ* and IL-4) were determined with murine cytokine ELISA kits (R&D systems, Minneapolis, MN). 

### 2.8. Statistical Analysis

 Student's *t*-test was employed in the statistical evaluation of the results.

## 3. Resuts

### 3.1. Immune Responses in Mice Immunized Intraperitoneally with OVA-Containing SucPG-Modified Liposomes

 Mice were administered intraperitoneally with OVA antigen, such as OVA alone (group I), polymer-(SucPG-) unmodified liposomes containing OVA (group II), and SucPG-modified liposomes containing OVA (group III), and antibodies against OVA were evaluated at 14 days after primary immunization.

**Figure 1:**
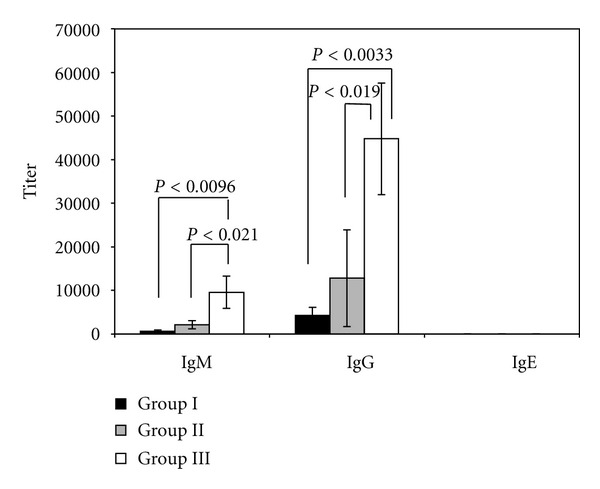
Serum anti-OVA antibody responses in mice administered OVA-containing SucPG-modified liposomes. Mice were immunized intraperitoneally with OVA alone (group I) or polymer-(SucPG-) unmodified liposomes entrapping OVA (group II) or SucPG-modified liposomes entrapping OVA (group III), and serum antibody titers were determined by ELISA on day 7 following secondary immunization. Results are expressed as the mean ± SEM in 5 different mice.

 As shown in Figure 1, in serum from mice receiving OVA alone (group I) and SucPG-unmodified liposomes containing OVA (group II), the production of anti-OVA IgM and IgG antibody was demonstrated, but not IgE antibody. On the other hand, higher-serum IgM and IgG activit against OVA was seen in the mice of group III. IgM and IgG antibody responses against OVA in group III were significantly higher than those in group I (IgM, *P* < 0.0096; IgG, *P* < 0.0033) and group II (IgM, *P* < 0.021; IgG, *P* < 0.019). The serum IgE antibody activities against OVA antigens were not detected in any mouse in group III. 

**Figure 2:**
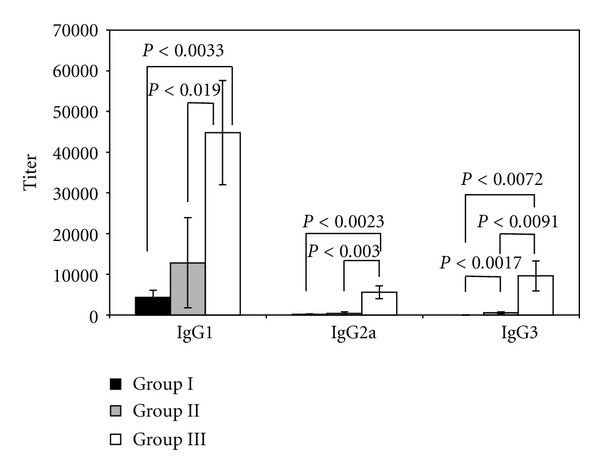
Profiles of OVA-specific IgG antibody subclasses in mice intraperitoneally immunized with OVA-containing SucPG-liposomes. Mice were immunized intraperitoneally with OVA alone (group I) or polymer-(SucPG-) unmodified liposomes entrapping OVA (group II) or SucPG-modified liposomes entrapping OVA (group III), and serum antibody titers were measured by ELISA on day 7 following secondary immunization. Results are expressed as the mean ± SEM in 5 different mice.

 Furthermore, serum Ab responses were characterized by analyzing the pattern of IgG subclasses present in sera from mice in groups I to III. As shown in Figure 2, only OVA-specific serum IgG1 Ab responses were demonstrated in the serum from mice immunized with OVA alone (group I). On the other hand, the induction of OVA-specific serum IgG1, IgG2a, and IgG3 antibody responses was demonstrated in sera from mice in groups II and III. In particular, the production of anti-OVA IgG1, IgG2a, and IgG3 antibody was significantly enhanced by the intraperitoneal administration of SucPG-modified liposomes containing OVA (group III) than by that of OVA-containing SucPG-unmodified liposomes (group II) (IgG1, *P* < 0.019; IgG2a, *P* < 0.003; IgG3, *P* < 0.0091).

### 3.2. Th1 and Th2 Cytokine Production by Spleen Cells from Mice Immunized Intraperitoneally with OVA-Containing SucPG-Modified Liposomes

**Figure 3:**
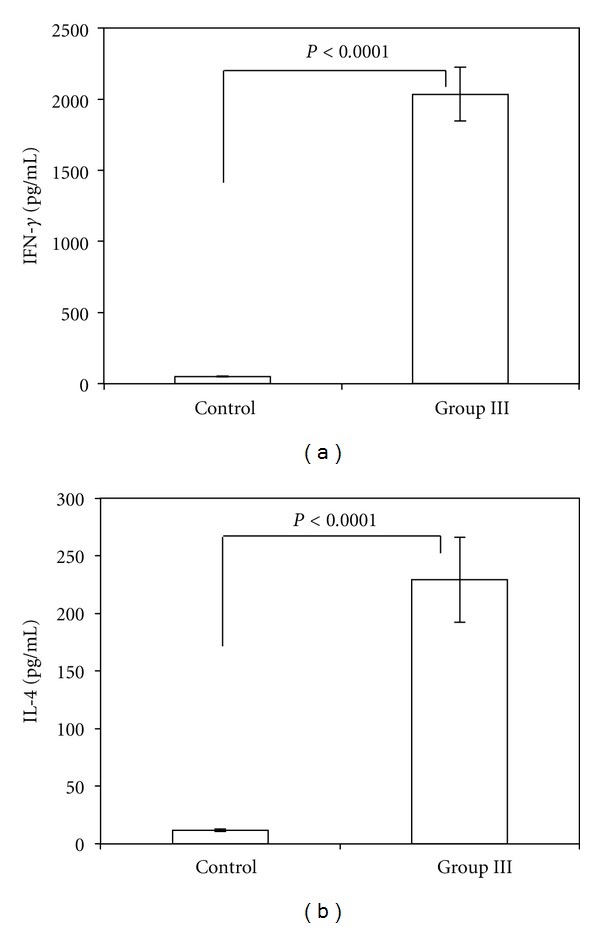
Th1 (IFN-*γ*) and Th2 (IL-4) cytokine secretion by spleen cells from mice after intraperitoneal administration of OVA-containing SucPG-modified liposomes. Spleen cells were harvested on day 7 after secondary immunization and cultured with OVA for 5 days. Subsequently, culture supernatants were collected for the analysis of cytokine production by ELISA. Values represent the mean ± SEM of cytokine production by spleen cells of mice in each group (nontreated control mice (Control) and OVA-containing SucPG-modified liposome-immunized mice (Group III)). (a) IFN-*γ*. (b) IL-4.

 The induction of OVA-specific serum IgG1, IgG2a, and IgG3 antibody responses by intraperitoneal immunization with OVA-containing SucPG-modified liposomes suggests efficient major histocompatibility complex presentation of the antigen leading to both humoral (IgG1) (Th2) and cell-mediated (IgG2a and IgG3) (Th1) responses (Figure 2). To characterize antigen-specific Th1 and Th2 responses, spleen cells were isolated from mice given SucPG-modified liposomes that entrap OVA (group III) and restimulated with OVA *in vitro*. Culture supernatants from OVA-stimulated spleen cells were then examined for the presence of Th1 and Th2 cytokines by ELISA. As shown in Figure 3, higher levels of both Th1 (IFN-*γ*) and Th2 (IL-4) cytokines were detected in the culture supernatant harvest from *in vitro* OVA-stimulated spleen cells from mice in group III than did spleen cells from nontreated control mice. 

### 3.3. Induction of IFN-*γ*- and IL-4-Specific mRNA in Spleen Cells from Mice Immunized Intraperitoneally with OVA-Containing SucPG-Modified Liposomes

**Figure 4:**
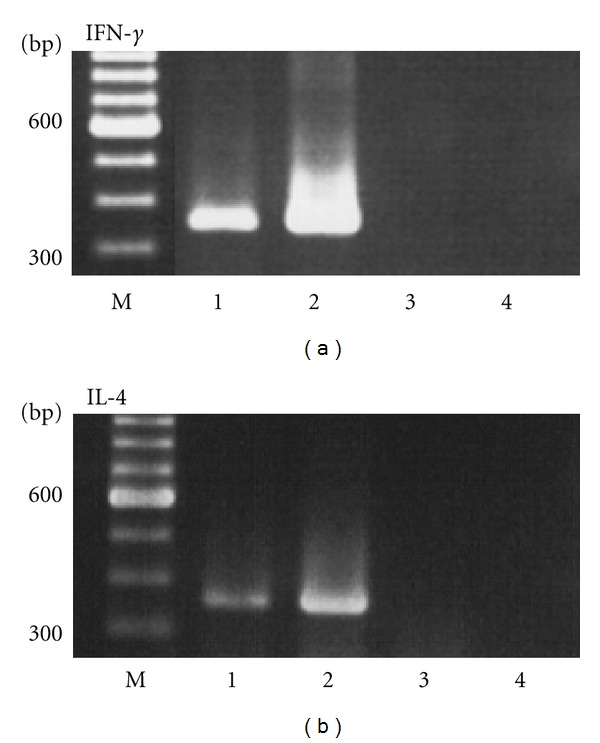
RT-PCR analysis of Th1 and Th2 cytokine-specific mRNA from spleen cells of mice immunized intraperitoneally with OVA-containing SucPG-modified liposomes. An identical experiment was repeated on three occasions with similar results. M, Marker (100 base ladder). Lane 1, Immunized mice. Lane 2, Positive control. Lane 3, Non-treated control mice. Lane 4, Negative control.

 The production of Th1-type (IFN-*γ*) and Th2-type (IL-4) cytokines by spleen cells from mice receiving intraperitoneal OVA-containing SucPG-modified liposomes following *in vitro* restimulation was confirmed (Figures 3(a) and 3(b)). To confirm this finding at molecular levels, Th1 and Th2 cytokine-specific RT-PCR was performed by using RNA samples extracted from spleen cells of mice intraperitoneally immunized with SucPG-modified liposomes containing OVA. Results are shown in Figure 4. mRNA for Th1-type cytokine, that is, IFN-*γ* (365 bp), and for Th2-type cytokine, that is, IL-4 (357 bp), were expressed in spleen cells from mice given OVA-containing SucPG-modified liposomes intraperitoneally (group III) (Figure 4, lane 1). However, neither IFN-*γ* mRNA nor IL-4 mRNA expression was detected in spleen cells from nontreated control mice.

## 4. Discussion

 Vaccines have played an important role in disease prevention and have made a substantial contribution to public health. Upon natural infection, it is known that the host responds by inducing both humoral and cellular immunities against the pathogen. However, most of the currently approved vaccines work by inducing humoral immunity [24–26]. For protection against viruses that are highly mutable and frequently escape from antibody-mediated immunity, humoral immunity is insufficient [27–30]. Consequently, the development of vaccines that induce cellular immunity is critical to novel vaccine strategies. Thus, the new adjuvants and carriers are essential to this aim. In particular, efficient vaccine delivery systems have been required for the achievement of protective immunity. Previously, it has been established that liposomes have the applicability as an adjuvant for use in vaccines [3, 31]. In addition, we have demonstrated that liposomes are an effective mucosal antigen-delivery vehicle for the induction of systemic and local immune responses [31, 32]. More recently, we have developed pH-sensitive fusogenic polymer, SucPG-modified liposomes [20]. These liposomes can deliver antigenic proteins into cytosol of dendritic cells [20], suggesting that SucPG-modified liposomes are able to induce both humoral (Th1) and cellular (Th2) immune responses against encapsulated antigens following the administration of the liposomes. In the present study, thus, we used the pH-sensitive fusogenic polymer (SucPG-) modified liposomes as antigen delivery vehicle for the vaccine and evaluated the ability of inducing Th1 and Th2 immune responses. 

 In this study, the intraperitoneal administration of OVA-containing SucPG-modified liposomes (group III) induced not only good serum IgM antibody responses directed against OVA, but also good serum IgG antibody responses directed against OVA (Figure 1). On the other hand, intraperitoneal immunization with polymer-unmodified liposomes containing OVA (group II) was also able to induce both serum IgM and IgG antibody responses. However, SucPG-modified liposomes containing OVA (group III) induced Ab responses (IgM and IgG) in mice greater than those induced by polymer-unmodified liposomes containing OVA (Figure 1). This indicates that SucPG-modified liposomes act as an effective adjuvant for potentiating IgM and IgG antibody responses in the serum when administered by intraperitoneal route and that the adjuvant properties of liposomes can be further enhanced by the inclusion of polymer such as SucPG in liposomes. Furthermore, we evaluated whether polymer-modified liposomes containing OVA induce IgE production, because IgE shows detrimental effects, such as allergy. In this study, intraperitoneal immunization with OVA-containing SucPG-modified liposomes did not elicit any IgE production against OVA (Figure 1), suggesting that polymer-modified liposomes might serve as a vaccine candidate without detrimental effects, such as allergic responses. 

 In the present study, it was shown that intraperitoneal immunization with SucPG-unmodified liposomes containing OVA (group II) and with OVA-containing SucPG-modified liposomes (group III) induced not only antigen-specific IgG1 response, but also IgG2a and IgG3 responses (Figure 2). However, anti-OVA IgG1, IgG2a, and IgG3 antibodies were significantly enhanced by SucPG-modified liposomes containing OVA in comparison to those induced after immunization with OVA-containing SucPG-unmodified liposomes (IgG1, *P* < 0.019; IgG2a, *P* < 0.003; IgG3, *P* < 0.0091) (Figure 2). IgG1 antibody is regulated by Th2-type cytokines, while IgG2a, and IgG3 antibodies are regulated by Th1-cytokines [33]. Thus, a higher induction of IgG1, IgG2a and IgG3 antibodies by SucPG-modified liposomes suggests efficient major histocompatibility complex class II presentation of the antigen leading to both humoral (Th2-type) (IgG1) and cell-mediated (Th1-type) (IgG2a and IgG3) responses. Actually, this was corroborated by the production of cytokines IFN-*γ* (Th1) and IL-4 (Th2) (Figure 3), as well as the induction of mRNA for IFN-*γ* and IL-4 by spleen cells of OVA-containing SucPG-modified liposome-immunized mice (Figure 4). 

 The most effective immune response against multiple pathogens involves a combination of both humoral and cellular components. This is even true for some obligate intracellular pathogens [34, 35]. In general, the immunogenicity of vaccines can be enhanced by the use of adjuvants such as alum (aluminum-based mineral salt) [36]. Alum has been used widely and successfully in many licensed vaccines and has a good track record of safety. It is considered the adjuvant of choice for vaccines against infectious diseases that can be prevented by the humoral immune response [37, 38]. However, some limitations of alum have been described. Notably, alum is a poor inducer of cell-mediated immunity and T helper 1 (Th1) responses, which are critical to novel vaccine strategies [38, 39]. Thus, there is need to develop new adjuvant formulations for use in the development of effective vaccines which can induce both humoral and cellular immunities against the pathogens. We have provided here evidence for induction of strong antigen-specific Th2 and Th1 immune responses. Therefore, a new immunizing method (vaccine) using pH-sensitive fusogenic polymer-modified liposomes, such as SucPG-modified liposomes, would clearly be valuable. To our knowledge, this study is the first to evaluate pH-sensitive fusogenic polymer-(SucPG-) modified liposomes for use as a vaccine carrier. This pH-sensitive fusogenic polymer-(SucPG-) modified liposome vaccine would be effective in eliciting protective immunity, thereby facilitating the eradication of the disease.

## 5. Conclusions

 In conclusion, the study was carried out to evaluate the usefulness of pH-sensitive fusogenic polymer-modified liposomes as a vaccine carrier. It was confirmed that pH-sensitive fusogenic polymer-(SucPG-) modified liposomes could serve effectively as an antigen delivery vehicle (vaccine carrier) for inducing immune responses and that both humoral (Th2-type) and cell-mediated (Th1-type) immunity were induced by intraperitoneal immunization with pH-sensitive fusogenic polymer-(SucPG-) modified liposomes. 

 In summary, it is expected to use pH-sensitive fusogenic polymer-(SucPG-) modified liposomes as vaccine delivery vehicles (vaccine carrier) for the induction of protective humoral and cell-mediated immunities. 

## Figures and Tables

**Table 1:** RT-PCR primers used in this study.

Gene	Primer sequences
IFN-*γ* sense	5′-TGCATCTTGGCTTTGCAGCTCTTCCTCATGGC-3′
IFN-*γ* antisense	5′-TGGACCTGTGGGTTGTTGACCTCAAACTTGGC-3′
Product: 365 bp GenBank M28621	
IL4 sense	5′-CCAGCTAGTTGTCATCCTGCTCTTCTTTCTCG-3′
IL4 antisense	5′-CAGTGATGTGGACTTGGACTCATTCATGGTGC-3′
Product: 357 bp GenBank M25892	
G3PDH sense	5′-ACCACAGTCCATGCCATCAC-3′
G3PDH antisense	5′-TCCACCACCCTGTTGCTGTA-3′
Product: 452 bp GenBank M32599	
